# Supplementary material for: Self-organization of modular network architecture by activity-dependent neuronal migration and outgrowth
Source: eLife. 2019 Sep 17;8:e47996. doi: 10.7554/eLife.47996 (PMC6783273; doi:10.7554/eLife.47996)
Supplement: Transparent reporting form [file elife-47996-transrepform.docx]

***eLife’s* transparent reporting form**

We encourage authors to provide detailed information *within their submission* to facilitate the interpretation and replication of experiments. Authors can upload supporting documentation to indicate the use of appropriate reporting guidelines for health-related research (see [EQUATOR Network](http://www.equator-network.org/%20)), life science research (see the [BioSharing Information Resource](https://biosharing.org/)), or the [ARRIVE guidelines](http://www.plosbiology.org/article/info:doi/10.1371/journal.pbio.1000412) for reporting work involving animal research. Where applicable, authors should refer to any relevant reporting standards documents in this form.

If you have any questions, please consult our Journal Policies and/or contact us: [editorial@elifesciences.org](mailto:editorial@elifesciences.org).

**Sample-size estimation**

- You should state whether an appropriate sample size was computed when the study was being designed
- You should state the statistical method of sample size computation and any required assumptions
- If no explicit power analysis was used, you should describe how you decided what sample (replicate) size (number) to use

Please outline where this information can be found within the submission (e.g., sections or figure legends), or explain why this information doesn’t apply to your submission:

We could not predict the distributions of the data and some of the analyses that were found relevant before the experiments. We therefore did not calculate group sizes before the experiments.

**Replicates**

- You should report how often each experiment was performed
- You should include a definition of biological versus technical replication
- The data obtained should be provided and sufficient information should be provided to indicate the number of independent biological and/or technical replicates
- If you encountered any outliers, you should describe how these were handled
- Criteria for exclusion/inclusion of data should be clearly stated
- High-throughput sequence data should be uploaded before submission, with a private link for reviewers provided (these are available from both GEO and ArrayExpress)

Please outline where this information can be found within the submission (e.g., sections or figure legends), or explain why this information doesn’t apply to your submission:

Information about replication is provided in the methods section, in the results section whenever statistical findings were reported and in the statistical tables.

**Statistical reporting**

- Statistical analysis methods should be described and justified
- Raw data should be presented in figures whenever informative to do so (typically when N per group is less than 10)
- For each experiment, you should identify the statistical tests used, exact values of N, definitions of center, methods of multiple test correction, and dispersion and precision measures (e.g., mean, median, SD, SEM, confidence intervals; and, for the major substantive results, a measure of effect size (e.g., Pearson's r, Cohen's d)
- Report exact p-values wherever possible alongside the summary statistics and 95% confidence intervals. These should be reported for all key questions and not only when the p-value is less than 0.05.

Please outline where this information can be found within the submission (e.g., sections or figure legends), or explain why this information doesn’t apply to your submission:

Information about statistical testing is provided in the methods section, in the results section whenever statistical findings were reported and in the statistical tables. The data points underlying Figs. 3-6 will be placed in a public repository when the manuscript is accepted for publication.

(For large datasets, or papers with a very large number of statistical tests, you may upload a single table file with tests, Ns, etc., with reference to sections in the manuscript.)

**Group allocation**

- Indicate how samples were allocated into experimental groups (in the case of clinical studies, please specify allocation to treatment method); if randomization was used, please also state if restricted randomization was applied
- Indicate if masking was used during group allocation, data collection and/or data analysis

Please outline where this information can be found within the submission (e.g., sections or figure legends), or explain why this information doesn’t apply to your submission:

Samples were assigned according to the experimental treatment of the cell cultures as

PKC^N^ (Control, no treatment), PKC^-^ (exposure to Goe6976), PKC^+^ (exposure to PMA). Individual networks were randomly assigned to a treatment.

This is described in the Methods section. There was no masking during group allocation.

**Additional data files (“source data”)**

- We encourage you to upload relevant additional data files, such as numerical data that are represented as a graph in a figure, or as a summary table
- Where provided, these should be in the most useful format, and they can be uploaded as “Source data” files linked to a main figure or table
- Include model definition files including the full list of parameters used
- Include code used for data analysis (e.g., R, MatLab)
- Avoid stating that data files are “available upon request”

Please indicate the figures or tables for which source data files have been provided:

Data and code to generate figures from them were uploaded as source data (Matlab code and *.mat files with the data) for:

Figure 3B, C, E, F, H, I: Morphology development, the remaining panels were derived from these data. Matlab code is provided.

Figure 4A-C, E.

Figure 5B-E, F. Figure 5G and H are derived from these data.

Figure 6A, B

Figure 2: Model code

The code for the model has not yet been uploaded but will be so after some additional commenting of the code.

Fig. 1 has no associated data.

Fig. 4D, 5A, 6C are for illustration purposes and have no associated statistical analyses.

Data and code to generate the statistical summary in Table 1 and Table 2 were provided.
